# Reducing the noise in signal detection of adverse drug reactions by standardizing the background: a pilot study on analyses of proportional reporting ratios-by-therapeutic area

**DOI:** 10.1007/s00228-014-1658-1

**Published:** 2014-03-07

**Authors:** Birgitta Grundmark, Lars Holmberg, Hans Garmo, Björn Zethelius

**Affiliations:** 1 Department of Surgical Sciences, Uppsala University, 75185 Uppsala, Sweden; 2 Department of Pharmacovigilance, Medical Products Agency, Uppsala, Sweden; 3 Regional Cancer Centre of the Uppsala-Orebro region, Uppsala University Hospital, Uppsala, Sweden; 4 Division of Cancer Studies, King’s College London, Medical School, London, UK; 5 Department of Public Health and Caring Sciences/Geriatrics, Uppsala University, Uppsala, Sweden; 6 Scientific Support/Epidemiology, Medical Products Agency, Uppsala, Sweden

**Keywords:** PRR, Adverse drug reactions, ADR, Signal detection, Pharmacovigilance, Disproportionality analysis

## Abstract

**Purpose:**

Disproportionality screening analysis is acknowledged as a tool for performing signal detection in databases of adverse drug reactions (ADRs), e.g., in the European Union (EU) Drug Authority setting. The purpose of this study was to explore the possibility of decreasing false-positive signals of disproportionate reporting (SDR) by calculating the proportional reporting ratio (PRR)-by-therapeutic area (TA), while still maintaining the ability to detect relevant SDRs.

**Methods:**

In the EudraVigilance (EV) Database, output from PRR calculated with a restricted TA comparator background was compared in detail to output from conventional authority-setting PRR calculations for four drugs: bicalutamide, abiraterone, metformin, and vildagliptin, within the TAs of prostate gland disease and type 2 diabetes mellitus.

**Results:**

ADR reports per investigated drug ranged from 2,400 to 50,000. The PRR-TA’s ability to detect true-positive SDRs (as acknowledged in approved labeling) was increased compared to the conventional PRR, and performed 8–31 % better than a recently proposed stricter EU-SDR definition. The PRR-TA removed false SDRs confounded by disease or disease spill-over by up to 63 %, while retaining/increasing the number of unclassified SDRs relevant for manual validation, and thereby improving the ratio between confounded SDRs (i.e., noise) and unclassified SDRs for all investigated drugs (possible signals).

**Conclusions:**

The performance of the PRR was improved by background restriction with the PRR-TA method; the number of false-positive SDRs decreased, and the ability to detect true-positive SDRs increased, improving the signal-to-noise ratio. Further development and validation of the method is needed within other TAs and databases, and for disproportionality analysis methods.

**Electronic supplementary material:**

The online version of this article (doi:10.1007/s00228-014-1658-1) contains supplementary material, which is available to authorized users.

## Introduction

Screening for statistical disproportionality in databases of adverse drug reactions (ADRs) is an accepted means for signal detection. To improve patient safety, signal detection is performed by drug companies, national competent authorities, and independent pharmacovigilance stakeholders. Several disproportionality methods are currently in use [1–5], but no gold standard has been established [6, 7]. They all share the ability to detect drug safety signals years earlier than traditional manual methods [8, 9]. Strengths, limitations, and differences between different pharmacovigilance signal detection methods including disproportionality methods have been analyzed and described previously [10], and are not the subject of this article.

Within the European Union (EU), the EudraVigilance (EV) database [11] is continuously screened using the proportional reporting ratio (PRR) method [1, 3, 12]. This method delivers signals of disproportionate reporting (SDRs)—reported ADR-drug combinations that (1) appear to a disproportionately high extent in a database (have an elevated PRR value), and (2) reach a case count above a pre-specified threshold. 

An SDR is merely a statistical finding that does not imply a causal relationship between a drug and the ADR [13]. Large amounts of SDRs are regularly delivered for manual validation within the EU system from continuously ongoing EV-PRR screening procedures. The PRR method is sensitive, but the majority of SDRs delivered represent noise, from e.g., statistical chance findings, artifacts, already acknowledged ADRs, confounding by disease, or by “disease spill-over”, i.e. aspects of the treated disease coded as an ADR. While a minority of false SDRs is easily dismissible as non-signals, most require expert knowledge of the drug and the disease to be rejected. Attempts to improve the method’s performance and thereby conserve manual expert resources within the EU have recently included altering the numerical threshold defining an SDR by increasing the required case count from the conventionally used 3–5 at present [14]. This, however, incurs an inevitable delay in the delivery of new SDRs and the detection of signals.

Improving the performance of disproportionality analysis methods by increasing the signal-to-noise ratio would thus be important. We attempted to reduce the background noise of false positive SDRs confounded by disease and disease spill-over within the EU signal detection system. The novel method investigated, hereafter called PRR-by-therapeutic area (PRR-TA), uses logically restricted comparator backgrounds for the PRR calculations in drugs for common therapeutic areas. Outputs were compared to conventional PRR calculations using the full EV database background. Comparisons were made using the conventional SDR-defining case count of 3, previously used within the common EU, compared to the recently suggested threshold of 5. Additionally, we compared the PRR-TA method’s ability to detect true-positive SDRs compared to the PRR. Further, the impact of the masking phenomenon [17], usually particularly evident in commercial databases [18], was explored.

Pilots analyzed were four different drugs for chronic disease: bicalutamide, abiraterone, metformin, and vildagliptin, within the two therapeutic areas of prostate gland disease and type 2 diabetes mellitus (T2DM).

## Material and methods

### Database

The ADR database used was the EV, used for signal detection purposes by drug authorities within the EU, including the European Medicines Agency. All serious ADRs reported worldwide for all drugs approved within the EU are mandated to be reported to the EV from all marketing authorization holders and EU drug authorities. The database is available online for drugs centrally approved within the EU [15]. The ADRs are coded using the MedDRA terminology [16]. New EU-PhV legislation that came into effect in July 2012 included alterations in reporting rules and definitions of ADRs. Therefore, ADR data until a cut-off date June 30, 2012, were included for analysis. All data used in the study are strictly on a group level; no individual case reports or identifiable patient data was used. Hence, according to applicable legislation, no approval from the ethics review board was needed for the study.

### Proportional reporting ratios, thresholds

Signals of disproportionate reporting (SDRs) for the four investigated pilot drugs were identified by calculating the proportional reporting ratio (PRR) [3] for all suspect drug-ADR combinations on a MedDRA preferred term (PT) level in EV (Supplementary Table 1). The SDRs were delivered from the PRR calculations using the a priori defined cut-off thresholds: both (a) a case count of ≥3 (SDR3) in EV, and (b) a lower 95 % confidence interval of the PRR of > 1.0, as were recommended within the EU at the time of the study initiation. A higher case count threshold of ≥5 cases (SDR5) for identifying an SDR, recently introduced in the EU/ EMA system, was analyzed for comparison. In this first step throughput screening, no stratification was performed, in line with EU standard procedure.

The method investigated, hereafter called 'PRR-by-therapeutic area (PRR-TA)' restricts the background for comparison (b, d in Supplementary Table 1) to consist of drugs from the two respective therapeutic areas instead of all drugs in the EV.

### Therapeutic areas

The two therapeutic areas chosen were (a) prostate gland disease, with hormonally active drugs used for prostate cancer (PrC) and benign prostate hyperplasia (BPH), and (b) T2DM, excluding insulin replacement therapy. The drugs selected within the selected TAs were: bicalutamide, abiraterone, metformin, and vildagliptin, representing different time windows of a drug life cycle; from long-term, well-established to newly marketed drugs.

### Prostate gland disease

The prostate gland disease drugs studied were the well-established bicalutamide (approved in the 1990s) and the more recently approved abiraterone (EU, 2011), both indicated for PrC.

The PRR-TA calculations for prostate gland disease used as background all drugs from ATC-codes L02AE, L02BB, L02BX, G04CA, and B, and were performed with a sequentially more restricted background, seen in models 1-4:

1. PRR: bicalutamide or abiraterone vs. the whole EV database
2. PRR-TA: bicalutamide or abiraterone vs. drugs indicated for PrC or BPH
3. PRR-TA: bicalutamide or abiraterone vs. drugs indicated for PrC only
4. Drug class PRR: bicalutamide vs. other anti-androgens

For abiraterone, being the only approved drug in its class, no model 4 calculation was applicable. As some BPH drugs have other indications than BPH and to decrease the effect of any off-label use, calculations in models 1–4 were performed both with and without restricting them in order to include reports specified as occurring in male patients (supplementary data).

### Type 2 diabetes mellitus

The T2DM example drugs studied were the well-established metformin (approved in the 1950s) and the more recently approved vildagliptin (EU 2008).

The PRR-TA calculations for T2DM used as background all drugs from ATC-code A10B and were performed with a sequentially more restricted background, seen in models 5-8 below. ADR reports on predefined, fixed-dose combination products were not included.

5. PRR: metformin or vildagliptin vs. the whole EV database.
6. PRR-TA: metformin or vildagliptin vs. non-insulin antidiabetic drugs
7. Drug class PRR: metformin vs. biguanides
8. Drug class PRR: vildagliptin vs. DPP4-inhibitors

### Acknowledged ADRs in the summary of product characteristics

As a reference of acknowledged (true-positive) ADRs, the EU Summaries of Product Characteristics (SPCs) as per July 2012 were used for vildagliptin and abiraterone, while SPCs for originator and generic products were combined to determine acknowledged ADRs for metformin and bicalutamide.

### Validation

We conducted a comparative analysis of the ability to detect acknowledged ADRs, i.e., positive controls of true-positive SDRs, and to reduce noise from SDRs confounded by disease and disease spill-over by using the PRR-TA SDR3 and the SDR5.

SDRs delivered in models 1-8, shown above, were independently evaluated and classified by experienced clinical experts in the field of oncology, diabetology, and pharmacovigilance as either

A. *True-positive SDRs* (i.e., acknowledged ADRs in the SPCs for each drug) or
B. Other SDRs representing terms not acknowledged as ADRs in the SPCs. These were in turn separated into:
C. *False positive SDRs confounded by indication or by indication spill-*over (i.e., irrelevant for further evaluation), and
D. *Unclassifiable SDRs*, relevant for further manual validation as possible new signals.

Results from the classification were compared and the differences obtained were resolved by consensus with reference to standard literature.

For bicalutamide, 950 different ADR terms had been reported (Supplementary Table 2), PRR calculation delivered 95 of these as SDR3s, and these were thus classified into groups of “A” or “B” and the “B” group was in turn separated into “C” or “D”.

Possible masking/de-masking of SDRs by using restricted backgrounds for the PRR calculations was evaluated by comparing true-positive SDRs, the “A”s, in the respective models. The concordance between the methods was hereby evaluated.

A comparative analysis of the ability of models 1-8 to deliver true-positive SDRs, “A”s, was performed, defining this ability as the percentage of acknowledged ADR terms in the SPC detected by the method. A similar analysis using the SDR5 in models 1 and 5, respectively, was also performed.

The number of delivered SDRs from the “C” (false positives) and “D” (unclassifiable and therefore, relevant) groups using models 1-8 was identified and compared. A similar comparison using the SDR5 in models 1 and 5, respectively, was also performed.

### Statistics

Statistical calculations of the PRR-TA were made using the open access tool “R” [19, 20], except for the analyses of the full EV database (models 1 and 5) for standard PRR using the EV Data WareHouse Tool.

Formal calculations of the different PRR methods’ accuracy, i.e., the “usual” two-by-two table to calculate the sensitivity, specificity, and positive predictive value, are not applicable.

Several SDRs often represent similar events and may point to one broader reference ADR term acknowledged as a true ADR in the respective SPCs, thus making detection of true positives ambiguous. Further, true-negative SDRs cannot be firmly established, as it is in this group that the new, not-yet-established ADRs are to be detected. Instead, we used a proxy measurement of the positive predictive properties of the methods’, calculated as a ratio between the number of false positive SDRs, “C”, and the unclassifiable, relevant SDRs, “D”, for models 1-8. With presumed ideal noise reduction by a decreased numerator “C” and preserved or increased denominator, “D” the C/D -ratio should approach zero.

## Results

The number of ADR reports for the four investigated drugs ranged from 2,400 for abiraterone to close to 50,000 for metformin (Supplementary Table 2). To compare: the total number of ADR reports for all drugs in the EV database was roughly 3.5 million. The average number of ADR reports per ADR term was 18 for metformin and 5 for bicalutamide, abiraterone, and vildagliptin, mirroring the on-the-market times.

### Conventional PRR calculations using the SDR3 and SDR5 thresholds

Relative frequencies of SDRs using 3 as the case count threshold (SDR3) among all reported ADR terms ranged from 10 % for bicalutamide (i.e. 95/950) to 17.9 % for vildagliptin (Supplementary Table 2)' the rest, 82–90 %, were thereby excluded from clinical evaluation. Increasing the SDR-defining case count to ≥5 (SDR5) reduced the number of SDRs for further validation and verification by between 14 % in the abiraterone (men only) analysis and 36 % for vildagliptin, also removing between 33–70 % of unclassified SDRs, potentially delaying detection and validation of important signals (Supplementary Table 2).

### PRR calculations by restricting the background of comparison; detection of acknowledged ADRs in SPCs, i.e. true-positive SDR

**Fig. 1:**
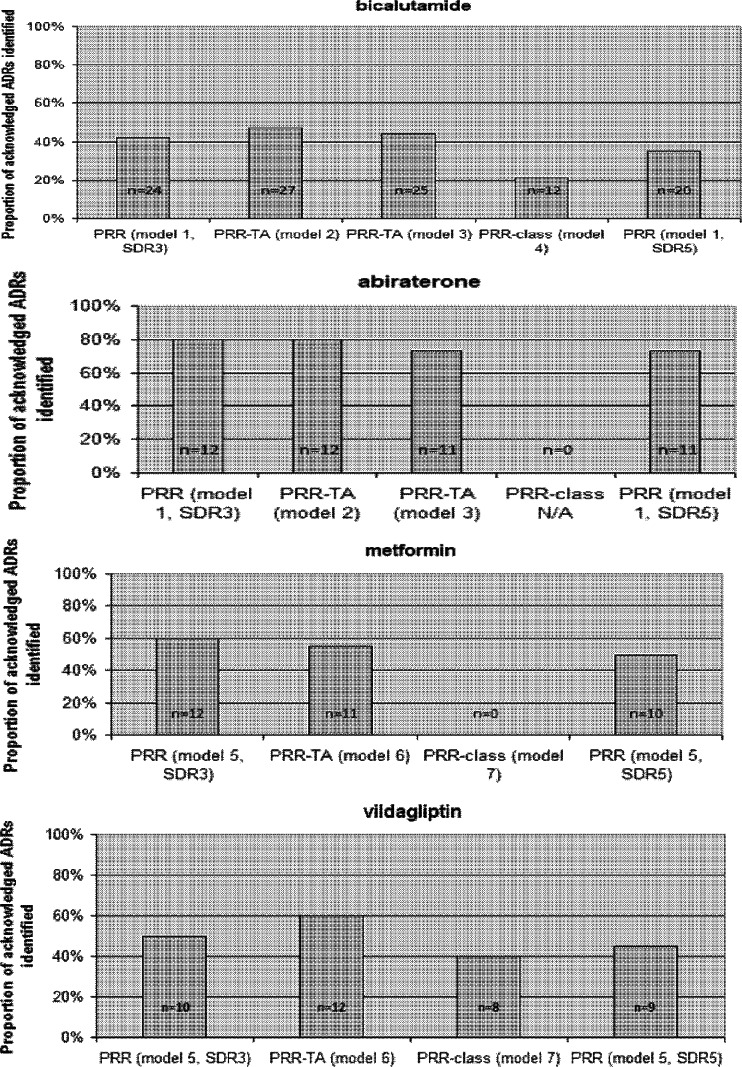
**a**-**d** The proportion of detected acknowledged ADRs, i.e., true-positive SDRs for bicalutamide (**a**), abiraterone (**b**), metformin (**c**), and vildagliptin (**d**) using from left to right for (**a**, **b**): the conventional PRR defining the SDR by a case count of ≥3 (model 1, SDR3); PRR-TA, prostate gland disease drugs (model 2, SDR3) ; PRR-TA prostate cancer drugs (model 3, SDR3); PRR-class (model 4, SDR3, not for abiraterone); and the conventional PRR defining the SDR by a case count of ≥5 (model 1, SDR5). (**c**, **d**) From left to right, the conventional PRR defining the SDR by a case count of ≥3 (SDR3); the PRR-TA(SDR3); PRR-class(SDR3); and the conventional PRR defining the SDR by a case count of ≥5 (SDR5)

The PRR-TA’s ability to detect and deliver true-positive SDRs compared to the conventional PRR method using SDR3 or SDR5 thresholds are presented in Fig. 1a-d. For bicalutamide, abiraterone, and vildagliptin, this ability was increased or unchanged (Fig. 1a, b, d). For metformin, the PRR-TA failed to deliver one of the twelve ADR terms delivered by the conventional PRR method (Fig. 1c).

Using the more strict SDR5 threshold (far right bar in Fig. 1a-d), led to a failure of the PRR to identify between 5–20 % of acknowledged ADRs as compared to using the SDR3 threshold. Applying the SDR5 threshold with the PRR failed to identify between 8–31 % compared to the PRR-TA method applying the SDR3.

Reducing the background further down to drug class resulted in a marked loss of ability to detect true-positive SDRs in the bicalutamide/anti-androgen (model 4) and vildagliptin/DPP4I (model 8) analyses, and an absence of ability to detect any true-positive SDRs in the metformin/biguanides analysis (model 7), indicating that models 4, 7, and 8 were not useful.

Analyses restricted to male gender for bicalutamide and abiraterone did not differ markedly compared to analyses including both genders; however, they appeared to perform less well (Supplementary Fig. 4–6).

The ability to detect true-positive SDRs by the PRRs methods using SDR 3 and SDR5 thresholds and the inter-method concordance using different backgrounds in models 1–8 were high for each drug investigated. A few true-positive SDRs were de-masked using the PRR-TA compared to conventional PRR. For all drugs, the ability to detect true-positive SDRs using the PRR-SDR5 was generally lower than for the PRR-SDR3 and the PRR-TAs.

### PRR calculation by restricting the background of comparison. Detection of SDRs not acknowledged as ADRs in the SPCs

**Fig. 2:**
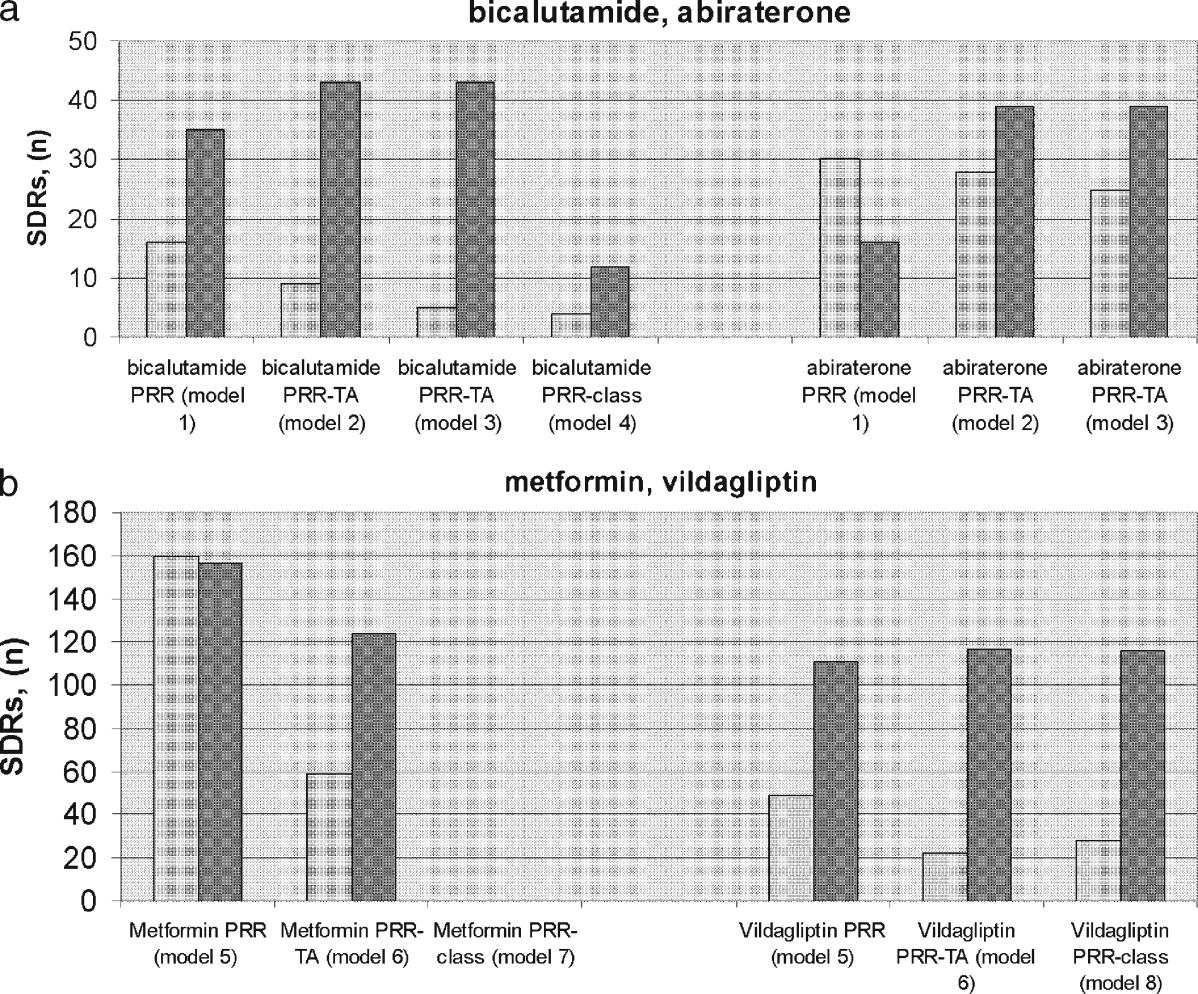
**a**-**b** The PRR, PRR-TAs, and the PRR class methods’ ability to detect and deliver SDRs not acknowledged as ADRs in the SPCs for each drug, either false-positive SDRs confounded by disease or disease spill-over (grey bars) or unclassified SDRs relevant for further manual validation (black bars); Fig 2**a**: bicalutamide and abiraterone, Fig 2**b**: metformin and vildagliptin analyses

Figure 2 a-b represents the PRR-TA method’s ability to detect and deliver SDRs *not* acknowledged as ADRs in the SPCs for each drug, either false-positive SDRs confounded by disease or disease spill-over (grey bars), or unclassified SDRs relevant for further manual validation (black bars).

The number of false-positive SDRs confounded by disease or disease spill-over, and thus less relevant for further evaluation, decreased when moving from the conventional PRR analysis to the PRR-TA (grey bars, from left to right in respective figures) for all drugs except for abiraterone analysis (men only; Fig. 2b).

The number of unclassified SDRs relevant for further manual validation, increased (black bars) when moving from the conventional PRR analysis to the PRR-TA (from left to right for each drug) for all drugs except for metformin.

Reducing the background further down to drug class delivered for metformin and bicalutamide (models 4, 7) few or no unclassified SDRs relevant for manual validation, while for vildagliptin (model 8), the numbers were maintained. Drug-class level PRR thus appeared less useful.

Analyses restricted to male gender for bicalutamide and abiraterone did not differ markedly compared to non-restricted analyses (Supplementary Fig. 4–5).

**Fig. 3:**
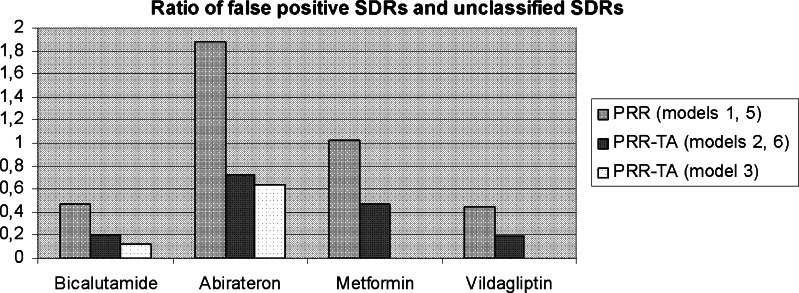
The ratio of false-positive SDRs confounded by indication or disease spill-over and unclassified SDRs relevant for further manual validation; the ratio should ideally be as close to zero as possible, with as few confounded SDRs as possible (numerator) delivered by the method in relation to the relevant SDRs (denominator). From left to right for each drug analysis: the ratios when analyzing by the conventional PRR, PRR-TA (model 2 and 6), and for bicalutamide and abiraterone, also the PRR-TA (model 3)

The ratio between false-positive SDRs confounded by indication or disease spill-over vs. unclassified SDRs relevant for further manual validation is visualized in Fig. 3. From left to right in the figure, the ratio for each of the drugs is consistently improved when decreasing the comparator background from the conventional PRR (SDR3) output to the PRR-TA.

Analyses restricting the background down to drug class (models 4, 7, 8) were not considered relevant to include in this analysis based on their poor performance regarding the ability to detect true-positive SDRs and remove false-positive SDRs.

## Discussion

### Main findings

Our study evaluates a novel approach of using the PRR method as the first step in a high throughput of disproportionality screening analysis—the PRR by therapeutic area (PRR-TA) using a background restriction, specifically in a drug authority pharmacovigilance standard setting. The evaluation of the PRR-TA is exemplified by drugs from areas of chronic disease: prostate gland disease and type 2 diabetes mellitus.

The PRR-TA performed better or equally well regarding its ability to detect true-positive SDRs and to reduce the noise in the form of false-positive SDRs, compared to the conventional PRR. A significant proportion of acknowledged ADRs were, however, not detected in any of the models often representing very general ADRs acknowledged for occurring with many drugs in the database, such as headache or nausea. The PRR-TA decreased the ratio between false-positive SDRs vs. unclassified SDRs relevant for further evaluation, i.e., improving the signal-to-noise relationship compared to the conventional PRR. Results from the most restricted backgrounds, the drug classes, were not satisfying for signal detection purposes, as their ability to detect true-positive SDRs was poor; this also confirmed the inherent feature of all signal detection methods in that the lack of detection of an SDR does not imply a lack of a causal relationship between a drug and a reported ADR.

The encouraging results when including drugs for the treatment of both BPH and PrC into one therapeutic area suggest that the indication for treatment of included drugs does not have to be identical in a restricted background database for the method to apply. It appears to suffice that the symptoms of the treated disease areas are largely coinciding.

The PRR-TA furthermore performed better for background noise reduction than the recently implemented method in the EU of restricting the number of SDRs delivered by increasing the threshold case count to ≥5 instead of ≥3. Analyses restricted by gender for the prostate gland disease analyses did not markedly improve the outcome, implying that gender restrictions may be less useful than restriction to therapeutic area, even in gender-specific drugs/therapeutic areas.

### Comparison with literature

A recent general guidance review on practical aspects on pharmacovigilance methodology briefly discusses a possible impact on disproportionate analyses from restricting the background or by stratification [10]. However, this is predominately suggested for the area of vaccines and pediatric drugs due to their particular use and target population. Among seven possible sources of improvements in signal detection suggested by others [21] is: “selection of appropriate control groups and restriction to subsets of people/reports”. It has been suggested that subgroups of a database could be used as a background for disproportionate analyses [22], by e.g., removing per-orals when analyzing injectables, chemotherapeutics when evaluating emesis for other drugs or, all ADR terms in the background that do not appear for a drug under investigation [22]. This has been exemplified with single-drug-ADR pairs, but no general analysis has been presented.

Few studies on the systematic analysis of results from background alterations for disproportionality methods have been published—these almost exclusively concerning the area of analyzing (pediatric-) vaccines [23–25]. Increased numbers of false negatives have been noted in such analyses [25], and only subsamples of SDRs (5–10 %) were analyzed in clinical detail. Stratification for age, gender, and/or alteration of background databases resulted in differences in the output from disproportionality analyses [23, 24] in vaccines, with low concordance in some cases [23] recommending combined analyses [24], and highlighting that *“*stratification likely increased efficiency*”* [23].

Published studies generally represent a statistics perspective on explorations of variants of restricted (vaccine) backgrounds or stratification in disproportionality analyses, again with only minor samples of the output analyzed in clinical detail [25]. The present study instead focuses on the clinical patient perspective, i.e., on the therapeutic area classifying each SDR in detail to determine its relevance. Furthermore, practical and generalizable conclusions drawn from vaccine signal detection studies are not applicable for long-term-use drugs, with vaccines being used on few occasions in a healthy, young population. Our study populations are in this respect more representative of long-term drug users in general, regarding variations in age, background morbidity, and drug administration forms.

The masking phenomenon from isolated drugs or ADRs may have a large impact on analyses, especially in commercial databases in which single drugs may constitute a large proportion of the reports [18, 24, 26–28]. We noted only sporadic cases of de-masking of acknowledged ADRs/SDRs in the PRR-TA compared to the PRR. The further evaluation of the phenomenon of de-masking by removing established ADR-drug associations from the background, as others have both hypothesized and performed [29, 30], could be another way of improving the screening performance.

Methods to measure and compare the general performance of disproportionality methods in ADR databases are under development, e.g., from OMOP collaboration [31], by using standard collections of positive and negative controls for drug-event-combinations (DECs). Such methods are not applicable for measuring the results of detailed analyses of individual drugs such as in our PRR-TA pilot study, as they are comprised only of a few selected controls per drug across a full database, rather than covering all ADRs acknowledged for a specific drug. The OMOP data were therefore not relevant for measurement in our study.

### Strengths and weaknesses of the PRR-TA

The PRR-TA reduced the background noise to a higher extent than was reached through restricting the number of signals by redefining, i.e., increasing the SDR threshold of the PRR from SDR3 to SDR5.

A strength of the PRR-TA is the possibility to avoid the inevitable delayed signal detection incurred by an increased case count threshold from 3–5, i.e., the delay while awaiting cases #4 and #5. This is especially relevant for orphan drugs or other drugs that are used less frequently.

An inherent weakness of the PRR-TA, shared with all disproportionate methods, is the poor ability to detect ADRs mimicking symptoms of the treated disease or opposite paradoxical reactions [32]. Such SDRs will likely be discarded early at the following step, i.e., the manual expert validation. Validation of a signal following a disproportionality analysis normally includes ascertaining reliable information on the background incidence of the suspected new ADR in the population at risk, i.e., in patients with the same disease without treatment with the drug in question. The PRR-TA partly incorporates this.

The PRR-TA thus represents a way of introducing established clinical knowledge early in the primary statistical disproportionality analysis, providing the manual evaluators the possibility to focus on relevant SDRs, with reduced noise from irrelevant SDRs.

### Clinical and scientific implications

This study explored new methods for signal detection, intending to decrease background noise while maintaining the ability to detect true signals. The PRR-TA method provides an opportunity to standardize data in order to improve the output in a large, general database.

Importantly, the therapeutic use in our study is the factor for clustering drugs rather than the ATC code, as the latter would presumably not be as sufficient for the reduction of SDRs representing confounding of disease and disease spill-over.

The PRR-TA would, if proven generalizable to other therapeutic areas, provide opportunity for a more cost-efficient use of manual expert resources in the ensuing signal validation step. Others have previously emphasized “the importance of minimizing the amount of false-positive signals (SDRs) that, if excessive, could detract from optimal pharmacovigilance activities” [24].

Advanced stratification or using advanced, complex statistical methods may provide an exaggerated confidence, i.e., “seduction bias” [33] as to what disproportionality methods may do. More complex analysis methods do not necessarily yield better output, especially if used at the expense of clinical expertise. Even if a signal-to-noise ratio or other features of a disproportionality analysis method are improved, these methods can indeed still be used only for screening purposes. The ensuing manual clinical expert evaluation is indispensable in determining whether a delivered SDR should be considered a signal or not.

The PRR-TA method balances well the method's complexity in relation to clinical knowledge in the areas exemplified.

### Unanswered questions and future research specified

The PRR-TA was performed in our study for drugs with a single approved indication in a very large general ADR database. Other drugs and their respective therapeutic areas would have to be analyzed before a wider use could be recommended. For drugs with more than one approved indication within diverse TAs, TA definitions also need further exploration. The method may be useful in other database settings or with other disproportionality methods, but this would similarly have to be validated before applying it in signal detection routine. If proven generalizable, the PRR-TA would have the potential to improve the output of screening methods currently used in the EU. At this point, our results would suffice to merit the use of the PRR-TA method in conjunction with conventional methods.

## Conclusions

The PRR-TA method, i.e., adapting the PRR method by therapeutic areas, suggests a potential to decrease the number of false-positive SDRs confounded by indication and indication spill-over. Further, the PRR-TA maintains the ability to detect true-positive SDRs in drugs for chronic diseases using the SDR threshold of three, i.e., without introducing inevitable delays of waiting for the fourth and fifth reports. We emphasize that exploring and validating the method’s applicability also in other treatment areas is needed to establish its position among present tools for signal detection, considering their different advantages and disadvantages. A conventional PRR method may not be replaced at present, rather the PRR-TA may be used in conjunction. If found to be generalizable into other therapeutic areas, this tool could increase the effectiveness of valuable manual validation resources.

## Electronic supplementary material

Below is the link to the electronic supplementary material.

ESM 1 (JPEG 270 kb)

High-resolution image (TIFF 721 kb)

ESM 2 (JPEG 126 kb)

High resolution image (TIFF 255 kb)

Supplementary table 1 (DOCX 13 kb)

Supplementary table 2 (DOCX 14 kb)

## References

[1] Finney DJ (1974). Systemic signalling of adverse reactions to drugs. Methods Inf Med.

[2] Bate A, Lindquist M, Edwards IR, Olsson S, Orre R, Lansner A, De Freitas RM (1998). A Bayesian neural network method for adverse drug reaction signal generation. Eur J Clin Pharmacol.

[3] Evans SJ, Waller PC, Davis S. Use of proportional reporting ratios (PRRs) for signal generation from spontaneous adverse drug reaction reports. Pharmacoepidemiol Drug Saf. 2001 Oct-Nov;10(6):483-6. 10.1002/pds.677 11828828

[4] Szarfman A, Machado SG, O'Neill RT (2002). Use of screening algorithms and computer systems to efficiently signal higher-than-expected combinations of drugs and events in the US FDA's spontaneous reports database. Drug Saf.

[5] Du Mouchel W, Smith T, Beasley R (2004). Association of asthma therapy and Churg-Strauss syndrome: an analysis of post-marketing surveillance. Clin Ther.

[6] van Puijenbroek EP, Bate A, Leufkens HG, Lindquist M, Orre R, Egberts AC. A comparison of measures of disproportionality for signal detection in spontaneous reporting systems for adverse drug reactions. Pharmacoepidemiol Drug Saf. 2002 Jan-Feb;11(1):3-10. 10.1002/pds.668 11998548

[7] Hochberg AM, Hauben M, Pearson RK, O'Hara DJ, Reisinger SJ, Goldsmith DI, Gould AL, Madigan D (2009). An evaluation of three signal-detection algorithms using a highly inclusive reference event database. Drug Saf.

[8] Hauben M, Reich L, Chung S (2004). Postmarketing surveillance of potentially fatal reactions to oncology drugs: potential utility of two signal-detection algorithms. Eur J Clin Pharmacol.

[9] Alvarez Y, Hidalgo A, Maignen F, Slattery J (2010). Validation of statistical signal detection procedures in eudravigilance post-authorization data: a retrospective evaluation of the potential for earlier signalling. Drug Saf.

[10] CIOMS. Practical Aspects of Signal Detection in Pharmacovigilance, Report of CIOMS Working Group VIII, CIOMS, (WHO), Geneva 2010.

[11] EMA, Eudravigilance, European database of suspected adverse drug reactions, http://eudravigilance.ema.europa.eu/highres.htm;.

[12] EudraVigilance Expert Working Group (EVEWG). Guideline of the use of statistical signal detection methods in the EudraVigilance data anlysis system. Doc Ref EMEA/106464/2006 rev 1, London: European Medicines Agency; June 2008.

[13] Hauben M, Aronson JK (2009). Defining 'signal' and its subtypes in pharmacovigilance based on a systematic review of previous definitions. Drug Saf.

[14] Slattery J, Alvarez Y, Hidalgo A. Choosing Thresholds for Statistical Signal Detection with the Proportional Reporting Ratio. Drug Saf. 2013 Jun 10.1007/s40264-013-0075-1 23754759

[15] EMA, Eudravigilance, European database of suspected adverse drug reactions, open access; http://adrreports.eu

[16] MSSO, http://www.meddramsso.com/

[17] Gould AL. Practical pharmacovigilance analysis strategies. Pharmacoepidemiol Drug Saf. 2003 Oct-Nov;12(7):559-74. 10.1002/pds.771 14558179

[18] Maignen F, Hauben M, Hung E, Van Holle L, Dogne J. Assessing the extent and impact of the masking effect of dispropoprtionality analyses on two spontaneous reporting systems databases. Pharmacoepidemiol Drug Saf 2013, [Ahead of Print], DOI:10.1002/pds.3529 10.1002/pds.3529 24243665

[19] Ihaka R, Gentleman RR (1996). A language for data analysis and graphics. J Comput Graph Stat.

[20] R statistics tool, http://www.r-project.org/

[21] Egberts TCG (2007). Signal detection: historical background, (Conference paper). Drug Saf.

[22] Gogolak VV. The effect of backgrounds in safety analysis: the impact of comparison cases on what you see. Pharmacoepidemiol Drug Saf. 2003 Apr-May;12(3):249-52. 10.1002/pds.823 12733479

[23] Woo EJ, Ball R, Burwen DR, Braun MM (2008). Effects of stratification on data mining in the US Vaccine Adverse Event Reporting System (VAERS). Drug Saf.

[24] Zeinoun Z, Seifert H, Verstraeten T (2009). Quantitative signal detection for vaccines: effects of stratification, background and masking on GlaxoSmithKline's spontaneous reports database. Hum Vaccin.

[25] de Bie S, Verhamme KM, Straus SM, Stricker BH, Sturkenboom MC (2012). Vaccine-based subgroup analysis in VigiBase: effect on sensitivity in paediatric signal detection. Drug Saf.

[26] Almenoff JS, Pattishall EN, Gibbs TG, DuMouchel W, Evans SJ, Yuen N. Novel statistical tools for monitoring the safety of marketed drugs. Clin Pharmacol Ther. 2007 Aug;82(2):157-66. Review 10.1038/sj.clpt.6100258 17538548

[27] Wang HW, Hochberg AM, Pearson RK, Hauben M (2010). An experimental investigation of masking in the US FDA adverse event reporting system database. Drug Saf.

[28] [28]Salvo F, Leborgne F, Thiessard F, Moore N, Begaud B, Pariente A. A Potential Event-Competition Bias in Safety Signal Detection: Results from a Spontaneous Reporting Research Database in France. Drug Saf. 2013. 10.1007/s40264-013-0063-5 23673817

[29] Pariente A, Avillach P, Salvo F, Thiessard F, Miremont-Salame G, Fourrier-Reglat A (2012). Effect of competition bias in safety signal generation: analysis of a research database of spontaneous reports in france. Drug Saf.

[30] Pariente A, Didailler M, Avillach P, Miremont-Salame G, Fourrier-Reglat A, Haramburu F, et al. A potential competition bias in the detection of safety signals from spontaneous reporting databases. Pharmacoepidemiol Drug Saf. 2010;19(11):1166-71. Epub 2010/09/18.) 10.1002/pds.2022 20848561

[31] Foundation for National Institutes of Health. Observational Medical Outcomes Partnership (online). http://omop.fnih.org/node/22.

[32] Smith SW, Hauben M, Aronson JK (2012). Paradoxical and bidirectional drug effects. Drug Saf.

[33] Hauben M, Patadia V, Gerrits C, Walsh L, Reich L (2005). Data mining in pharmacovigilance: the need for a balanced perspective. Drug Saf.

