# Supplementary figures and images for: Reducing the noise in signal detection of adverse drug reactions by standardizing the background: a pilot study on analyses of proportional reporting ratios-by-therapeutic area
Source: Eur J Clin Pharmacol. 2014 Mar 7;70(5):627–35. doi: 10.1007/s00228-014-1658-1 (PMC3978377; doi:10.1007/s00228-014-1658-1)

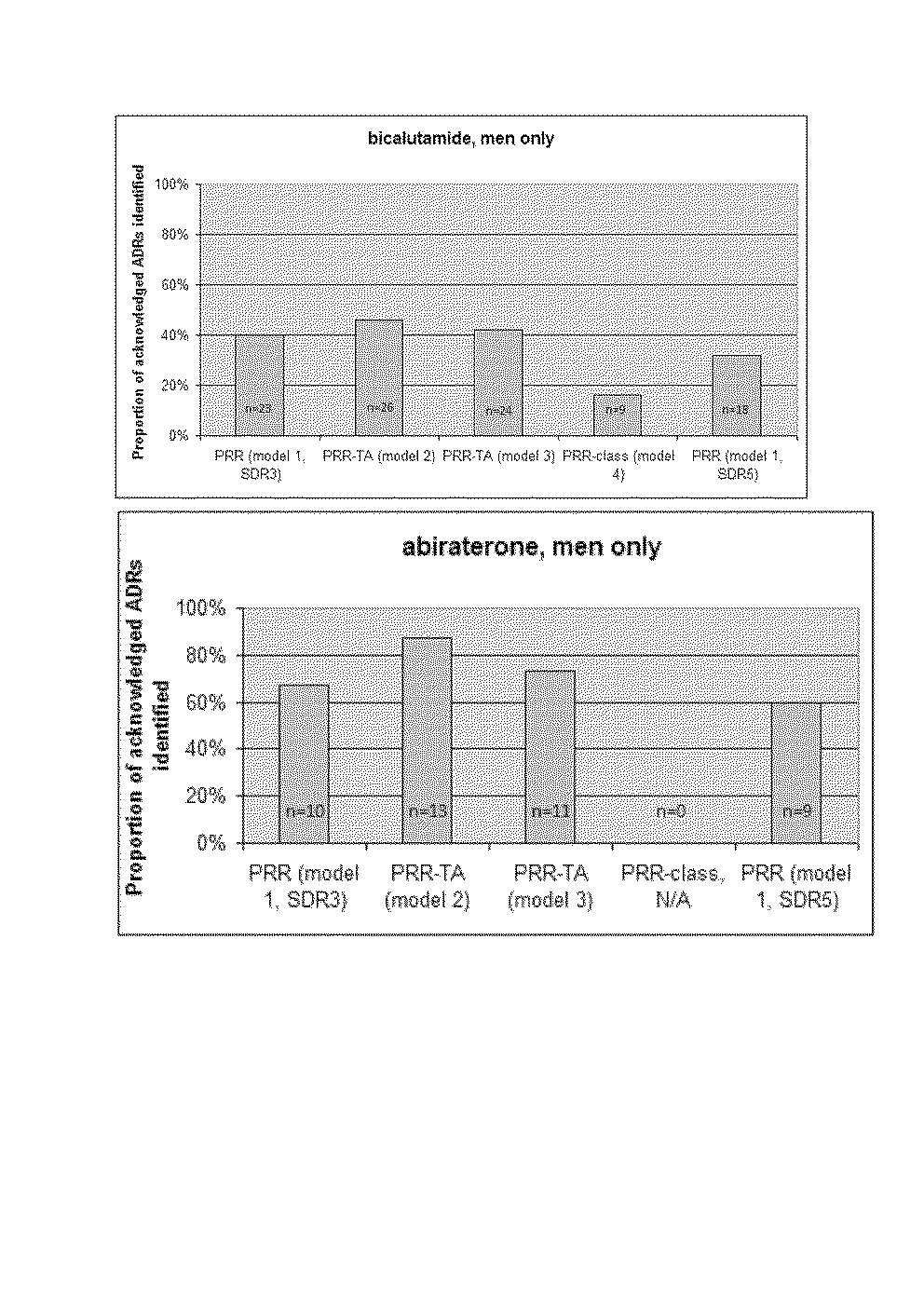

Supplement: Supplementary file 1 — (JPEG 270 kb) [file 228_2014_1658_Fig4_ESM.jpg]

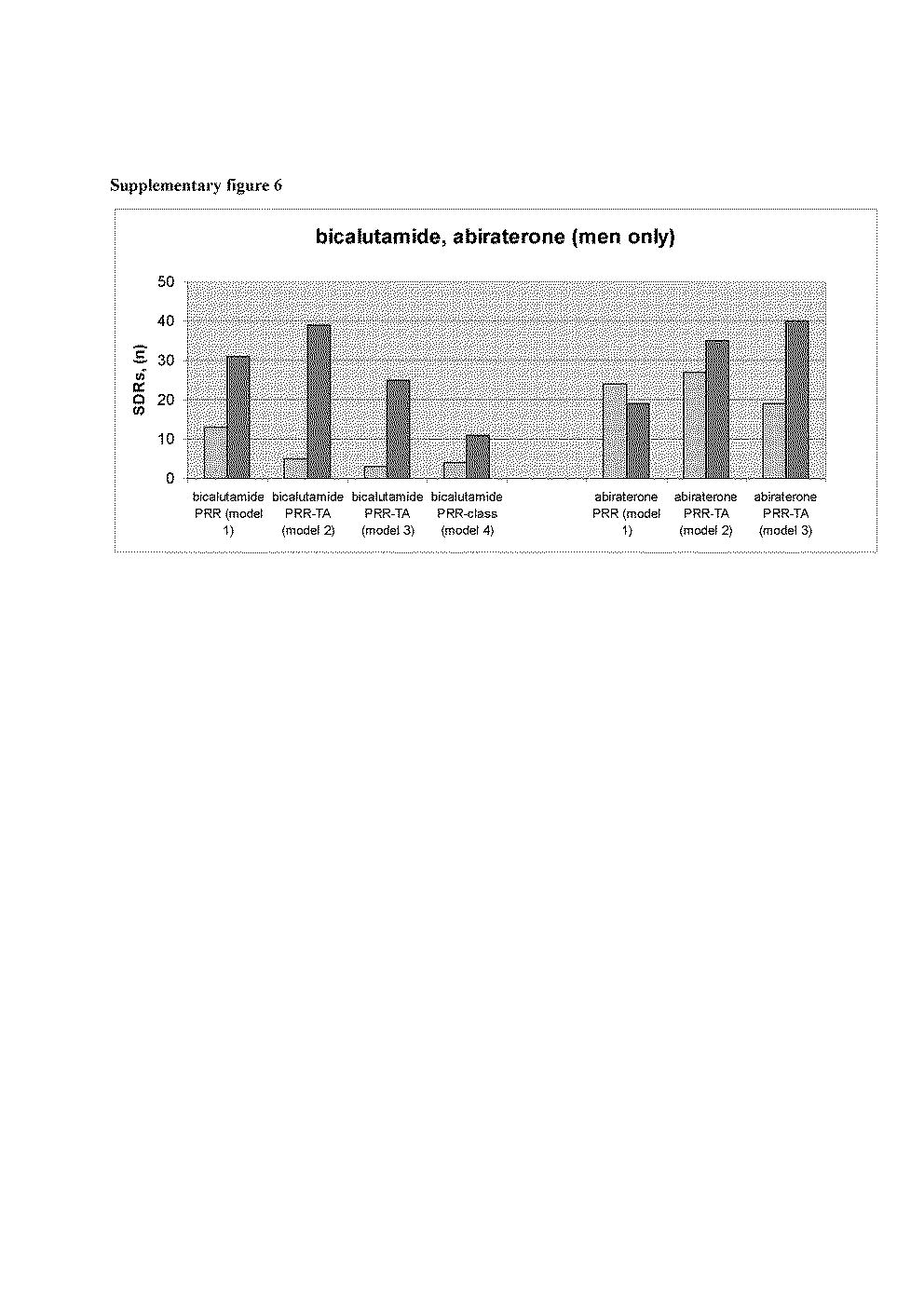

Supplement: Supplementary file 3 — (JPEG 126 kb) [file 228_2014_1658_Fig5_ESM.jpg]
