# Supplementary material for: Reducing the noise in signal detection of adverse drug reactions by standardizing the background: a pilot study on analyses of proportional reporting ratios-by-therapeutic area
Source: Eur J Clin Pharmacol. 2014 Mar 7;70(5):627–35. doi: 10.1007/s00228-014-1658-1 (PMC3978377; doi:10.1007/s00228-014-1658-1)
Supplement: Supplementary file 5 — (DOCX 13 kb) [file 228_2014_1658_MOESM3_ESM.docx]

**Supplementary table 1** Calculation of the PRR for all drug-ADR combinations equals [a/(a+c)]/[b/(b+d)]

|  | Drug under investigation | All other drugs |
| --- | --- | --- |
| Reaction of interest | a | b |
| All other reactions | c | d |
|  | a+c | b+d |
