# Supplementary material for: Reducing the noise in signal detection of adverse drug reactions by standardizing the background: a pilot study on analyses of proportional reporting ratios-by-therapeutic area
Source: Eur J Clin Pharmacol. 2014 Mar 7;70(5):627–35. doi: 10.1007/s00228-014-1658-1 (PMC3978377; doi:10.1007/s00228-014-1658-1)
Supplement: Supplementary file 6 — (DOCX 14 kb) [file 228_2014_1658_MOESM4_ESM.docx]

**Supplementary table 2** Number of adverse drug reaction reports, reported ADR terms and SDRs detected using the conventional PRR method; number of SDRs not delivered when increasing the threshold of the SDR-defining case count number from 3 to 5; number and fraction of the above of non-disease related SDRs relevant for manual validation.

|  | bicalutamide | | abiraterone | | metformin | vildagliptin |
| --- | --- | --- | --- | --- | --- | --- |
| Gender | unspecified | men only | unspecified | men only | unspecified | unspecified |
| ADR* reports in EV; n | 5 161 | 5 055 | 2 405 | 2 335 | 49 719 | 5 551 |
| ADR terms in EV**; n | 950 | 939 | 492 | 488 | 2 667 | 1 060 |
| SDR3s***; n,  ( % of all ADR terms) | 95 (10.0) | 79 (8.4) | 70 (14.2) | 63 (12.9) | 371 (13.9) | 190 (17.9) |
| SDR5s****; n | 66 | 55 | 58 | 54 | 304 | 120 |
| SDRs** undetected when moving from SDR3 to SDR5; n | 29 | 24 | 12 | 9 | 67 | 70 |
| SDRs relevant for manual evaluation but undetected when moving from SDR3 to SDR5; n, (%) | 13 (44.8) | 13 (54.1) | 5 (41.7) | 3 (33.3) | 44 (70.0) | 46 (65.7) |

* ADR: Adverse Drug Reaction,

** EV: EudraVigilance database

*** SDR3: signal of disproportionate reporting using case count of ≥3

**** SDR5: signal of disproportionate reporting using case count of ≥5
